# Abortive Lytic Reactivation of KSHV in CBF1/CSL Deficient Human B Cell Lines

**DOI:** 10.1371/journal.ppat.1003336

**Published:** 2013-05-16

**Authors:** Barbara A. Scholz, Marie L. Harth-Hertle, Georg Malterer, Juergen Haas, Joachim Ellwart, Thomas F. Schulz, Bettina Kempkes

**Affiliations:** 1 Department of Gene Vectors, Helmholtz Zentrum München, German Research Center for Environmental Health, Munich, Germany; 2 Division of Pathway Medicine, University of Edinburgh, Edinburgh, United Kingdom; 3 Institute of Molecular Immunology, Helmholtz Zentrum München, German Research Center for Environmental Health, Munich, Germany; 4 Institute of Virology, Hannover Medical School, Hannover, Germany; Baylor College of Medicine, United States of America

## Abstract

Since Kaposi's sarcoma associated herpesvirus (KSHV) establishes a persistent infection in human B cells, B cells are a critical compartment for viral pathogenesis. RTA, the replication and transcription activator of KSHV, can either directly bind to DNA or use cellular DNA binding factors including CBF1/CSL as DNA adaptors. In addition, the viral factors LANA1 and vIRF4 are known to bind to CBF1/CSL and modulate RTA activity. To analyze the contribution of CBF1/CSL to reactivation in human B cells, we have successfully infected DG75 and DG75 CBF1/CSL knock-out cell lines with recombinant KSHV.219 and selected for viral maintenance by selective medium. Both lines maintained the virus irrespective of their CBF1/CSL status. Viral reactivation could be initiated in both B cell lines but viral genome replication was attenuated in CBF1/CSL deficient lines, which also failed to produce detectable levels of infectious virus. Induction of immediate early, early and late viral genes was impaired in CBF1/CSL deficient cells at multiple stages of the reactivation process but could be restored to wild-type levels by reintroduction of CBF1/CSL. To identify additional viral RTA target genes, which are directly controlled by CBF1/CSL, we analyzed promoters of a selected subset of viral genes. We show that the induction of the late viral genes ORF29a and ORF65 by RTA is strongly enhanced by CBF1/CSL. Orthologs of ORF29a in other herpesviruses are part of the terminase complex required for viral packaging. ORF65 encodes the small capsid protein essential for capsid shell assembly. Our study demonstrates for the first time that in human B cells viral replication can be initiated in the absence of CBF1/CSL but the reactivation process is severely attenuated at all stages and does not lead to virion production. Thus, CBF1/CSL acts as a global hub which is used by the virus to coordinate the lytic cascade.

## Author Summary

Kaposi's sarcoma associated herpesvirus (KSHV) establishes a life-long persistent infection in B cells, which constitute the viral reservoir for reactivation and production of progeny virus. Viral reactivation is associated with multiple AIDS related malignancies including Kaposi's sarcoma, an endothelial tumor, and two B cell lymphoproliferative malignancies, the primary effusion lymphoma and the multicentric Castleman's disease. CBF1/CSL is a cellular DNA binding protein that can recruit transactivators or repressors to regulatory sites in the viral and cellular genome. The replication and transcription activator (RTA) plays an essential role in the switch between latency and lytic reactivation. RTA can either bind to DNA directly or is recruited to DNA via anchor proteins like CBF1/CSL and activates transcription. In this study we used a novel cell culture model to analyze the contribution of the CBF1/CSL protein to the process of viral reactivation in human B cells. Two isogenic CBF1/CSL proficient or deficient B cell lines were latently infected with recombinant KSHV. Lytic viral gene expression, viral replication and virus production were compared. Our results suggest that viral lytic gene expression is severely attenuated but not abolished at multiple stages before and after the onset of lytic replication while virus production is below detection levels in CBF1/CSL deficient B cells.

## Introduction

Kaposi's sarcoma associated herpesvirus (KSHV) establishes a persistent infection in the human host. Infected human B cells in the circulation of the infected host are likely to constitute a major latent reservoir, from where KSHV can reactivate and spread. In addition, the strong association of KSHV with primary effusion lymphoma (PEL) and the plasma cell variant of multicentric Castleman's disease strongly suggests a causative role of the virus in the pathogenesis of these B cell diseases [1]–[4]. Thus, human B cells are likely to comprise a very important compartment for the persistent KSHV infection. The study of latent and lytic life cycle in B cells has been the focus of many studies in the past.

The replication and transcription activator (RTA) is a KSHV immediate early protein that activates a broad spectrum of lytic viral genes and thereby induces lytic reactivation. RTA can either directly bind to RTA-responsive elements or use cellular DNA binding factors like Ap-1, C/EBP-α, Oct-1 and CBF1/CSL as adaptors to DNA as reviewed [5]. The DNA binding factor CBF1/CSL (C-promoter binding factor, Suppressor of hairless, and Lag1 also designated CSL or RBP-J*κ*) is a highly conserved ubiquitously expressed protein and the major effector of Notch receptor signaling. In the following we will use the term CBF1 for this protein. It can serve as adaptor for transactivators but also can recruit corepressor complexes and thereby silence gene expression [6], [7]. Two general experimental strategies focussing on the cellular interaction partner or the viral genome have been used to study the functional implications of the RTA-CBF1 interaction. Initially, infection and virus production of CBF1 proficient and deficient murine fibroblasts was studied in isogenic systems. These studies suggested lytic reactivation is blocked while establishment of latency is not impaired in cells lacking CBF1 [8], [9]. Alternatively, computational prediction of potential CBF1 binding sites in the viral genome was followed by biochemical and mutational analysis of the respective viral promoters in promoter reporter assays or in the context of the viral genome by recombinant virus technologies. In addition, activation of viral targets by activated Notch defined further sets of CBF1 dependent promoters. In summary, these studies convincingly showed, that CBF1 contributes to the activation of ORF6, ORF8, ORF19, ORF47, ORF50, ORF57, ORF59, ORF61, ORF70, K2, K5, K6, K8, K14 and PAN as well as the latency transcript cluster (summarized and referenced in Table 1). Importantly, these genes control multiple stages in the viral latent and lytic life cycle. The relevance of the contribution of CBF1 to target gene control varied among the individual genes that were studied. Thus, the question remained to which extent the activity of CBF1 contributes to the process of viral reactivation and virus production in human B cells.

**Table 1:** CBF1 regulated KSHV genes.

Gene^a^	Function^b^	References
	**Viral DNA replication**	
ORF6	ssDNA binding protein	[8]
K8/K-bZIP	Origin binding protein	[52]
ORF57/MTA	Post-transcriptional regulator of gene expression	[53] [8]
ORF70	Thymidylat-synthase	[54]
ORF59	DNA replication processivity factor	[41]
ORF61	Ribonucleotide reductase	[52]
ORF21^c^	Thymidine kinase	[55]
	**Virus assembly**	
ORF8/gB	*Glycoprotein B*	[56]
ORF19	Tegumentprotein	[54]
ORF47	Glycoprotein L	[57] [54]
ORF65	Capsomer-interacting protein	shown in this study
ORF29a	Packaging protein	shown in this study
	**Modulation of Immune response**	
K5/vMIR2^c^	viral modulator of immune recognition	[58]
K6/vMIP-I	viral macrophage inflammatory protein-II	[53]
K2/vIL-6	viral Interleukin-6	[58]
	**Cell cycle and apoptosis**	
LTi (latency transcript cluster)^d^	ORF71/Flice inhibitory protein	[59]
	ORF72/cyclin-D homologue	[59]
	ORF73/Latency associated nuclear antigen	[60]
K14/ORF74 (bicistronic Promoter)^e^	K14/OX-2-Glycoprotein homologue	[36]
	ORF74/viral G-protein coupled receptor	[36]
	**Lytic Transactivator**	
ORF50/RTA	replication and transcription activator	[9]
	**unknown**	
nut-1/PAN	-	[8]

a These KSHV genes contain one or more functional CBF1 binding sites.

b Gene functions are taken from Jenner and Boshoff (Jenner and Boshoff, 2002).

c These genes contain functional CBF1 binding sites, but activation in response to RTA still needs to be addressed.

d The latency transcript cluster encodes for the latent genes ORF71, ORF72 and ORF73.

e The bicistronic K14/ORF74 transcript encodes for ORF74 and K14.

*In vitro* a broad spectrum of cell lines derived from diverse tissues can be infected with KSHV but human B cells and B cell lines appeared to be refractory to infection [4], [10]–[13]. Most recently, the productive infection of primary B cells could be accomplished but did not lead to long term proliferation of the infected cells [14]–[17]. After infection of the lymphoblastoid B cell line BJAB with cell-associated recombinant virus and cultivation of these infected BJAB cells in selective medium stable latently infected cultures were generated. However, viral reactivation was attenuated and virion production was almost blocked in these human B cells, rendering them unsuitable to address the role of CBF1 in lytic reactivation [18].

The goal of this study was to analyze the contribution of CBF1 to viral lytic reactivation in KSHV infected human B cells. To this end, we have infected isogenic CBF1 deficient and proficient human B cell lines with recombinant KSHV and compared them for viral reactivation and virus production. Our results indicate that viral reactivation is initiated in both CBF1 proficient and deficient B cells but viral gene expression is severely attenuated and virus production is below the detection level in the absence of CBF1. In addition to the viral genes already known to be controlled by CBF1, we could identify two further direct CBF1 dependent RTA target genes, ORF29a and ORF65.

## Results

### CBF1 deficient and proficient human B cell lines can be infected with recombinant KSHV, express viral latent antigens and maintain the viral genome in the presence of selective cell culture medium

**Figure 1:**
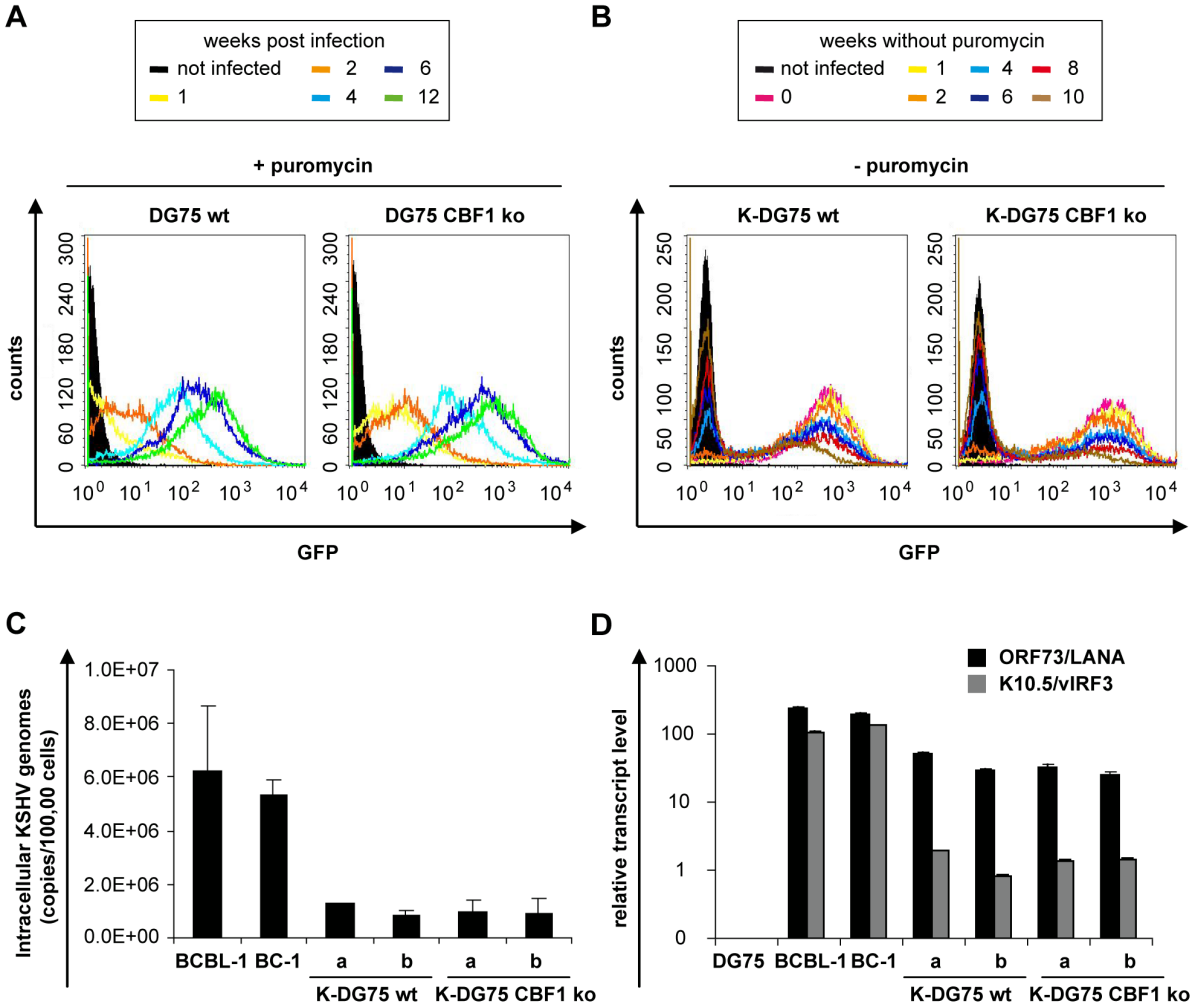
CBF1 proficient and deficient human B cell lines infected with recombinant KSHV maintain the viral genome and express viral latent genes when cultivated in selective media. DG75 wt and DG75 CBF1 ko cells were infected with recombinant rKSHV.219 virus at a MOI of 5. (A) GFP expression of DG75 wt and DG75 CBF1 ko cells infected with rKSHV.219 was analyzed by flow cytometry 1, 2, 4, 6, and 12 weeks post infection. Three independent experiments were performed and one representative experiment is shown. (B) rKSHV.219 infected DG75 wt (K-DG75 wt) and DG75 CBF1 ko (K-DG75 CBF1 ko) cells were transferred to puromycin free medium and analyzed for GFP expression by flow cytometry after 0, 1, 2, 4, 6, 8, and 10 weeks. Two independent experiments were performed and one representative experiment is shown. (C) Genomic DNA from BCBL-1, BC-1 and two independently generated K-DG75 wt and K-DG75 CBF1 ko cell lines (designated a and b) was used to quantify intracellular KSHV copy numbers by real-time PCR using primers specific for the ORF50 promoter and normalized to β-actin gene fragments. Results are presented as mean values calculated from 2 independent experiments. (D) Transcript levels of ORF73/LANA and K10.5/vIRF3 were determined by real-time RT-PCR analyses of DG75, BCBL-1, BC-1, K-DG75 wt and K-DG75 CBF1 ko cell lines. Results were normalized to β-actin transcript levels and are presented as mean values calculated from 2 independent experiments.

In order to analyze the contribution of CBF1 to viral reactivation in KSHV infected B cells we used a CBF1 negative human somatic B cell line. This cell line had been generated previously by gene targeting of the cellular CBF1 gene in the EBV negative Burkitt's lymphoma B cell line DG75 [19]. For infection of CBF1 proficient parental (DG75 wt) and CBF1 deficient (DG75 CBF1 ko) DG75 cells we used cell culture supernatants of the recombinant virus rKSHV.219 produced in Vero cells. The recombinant virus rKSHV.219 is a derivative of the KSHV strain obtained from JSC-1 cells [20]. rKSHV.219 encodes for red fluorescent protein (RFP) under the control of a CBF1 independent fragment of the KSHV early lytic PAN promoter, constitutively expresses green fluorescent protein (GFP), and carries a puromycin resistance cassette as a selectable marker. DG75 wt and DG75 CBF1 ko cells were infected with rKSHV.219 at a multiplicity of infection (MOI) of factor 5. GFP expression was monitored by flow cytometry during a time course of 12 weeks. A GFP positive cell population could be detected 1 week post infection. The intensity of the GFP signals recorded by flow cytometry was initially weak but increased over time in both, CBF1 proficient and deficient B cell lines (Fig. 1A). The results suggest that stable homogenous KSHV infected B cell cultures are generated within 4–6 weeks post infection irrespective of the CBF1 status. High GFP expression remained stable as long as the cultures were maintained in media containing puromycin (Fig. 1A). If the established rKSHV.219 infected DG75 wt and DG75 CBF1 ko cell lines (K-DG75 wt and K-DG75 CBF1 ko) were cultivated in the absence of puromycin a GFP negative population developed. Only 30% of CBF1 proficient and 22% of CBF1 deficient B cells were still GFP positive after they had been cultivated in puromycin free media for 10 weeks (Fig. 1B). Hence, viral maintenance required selection in both B cell lines. Viral genome copy numbers of established K-DG75 wt and K-DG75 CBF1 ko cell lines were determined and compared to data obtained from two independent KSHV infected PEL cell lines, BCBL-1 and BC-1. As expected 50 to 60 intracellular genomes were detected in BCBL-1 and BC-1 cells [21], [22]. K-DG75 wt and CBF1 ko cells carried approximately 10 viral copies per cell (Fig. 1C). Both K-DG75 cell lines, CBF1 proficient and deficient, expressed the KSHV latent marker genes ORF73/LANA and K10.5/vIRF3 at similar levels (Fig. 1D). ORF73/LANA is consistently expressed in latently infected cells of different tissue origin and required to maintain episomal viral genomes during cell division [23]–[25]. Latent expression of K10.5/vIRF3 is only seen in KSHV infected B cells [26]. The relative transcript levels of both viral genes were lower than in BCBL-1 and BC-1 cells but readily detectable. These distinct expression levels might partially reflect the lower viral genome copy numbers in the K-DG75 cell lines compared to BCBL-1 and BC-1.

**Figure 2:**
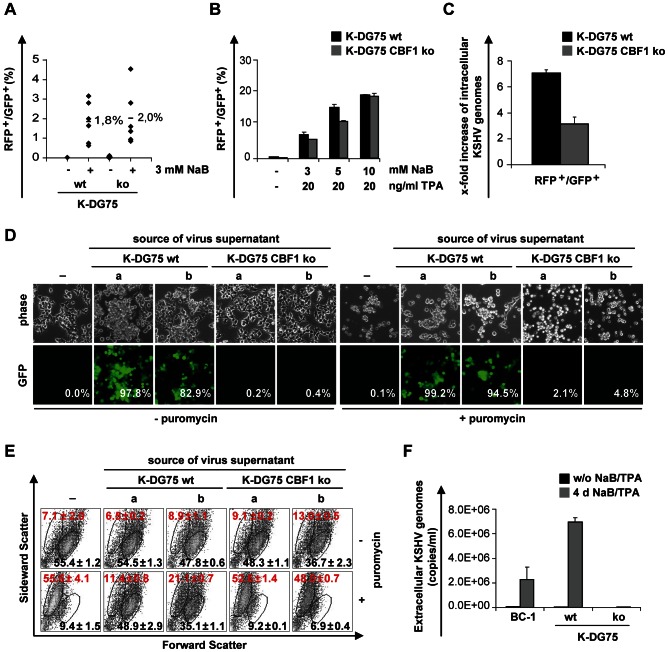
CBF1 deficient KSHV infected B cells initiate lytic reactivation but fail to produce detectable levels of infectious virus. (A) Six independent K-DG75 wt and K-DG75 CBF1 ko cell lines were treated with 3 mM NaB for 32 h and analyzed for GFP and RFP expression by flow cytometry. (B) K-DG75 wt and K-DG75 CBF1 ko cells were treated with increasing amounts of NaB and TPA for 32 h and analyzed for GFP and RFP expression. (A and B) Results are displayed as percentage of lytically reactivated cells as determined by RFP expression and are given as mean values of 2 independent experiments. For the following experiments (C, D, E and F) K-DG75 wt and K-DG75 CBF1 ko cells were treated with 10 mM NaB and 20 ng/ml TPA. (C) RFP^−^/GFP^+^ and RFP^+^/GFP^+^ cells were separated by cell sorting 32 h post induction of the lytic cycle by TPA/NaB treatment. Genomic DNA of both populations was analyzed for viral copy numbers by real-time PCR. The increase of viral copy numbers of lytically reactivated RFP^+^/GFP^+^ cells compared to latent RFP^−^/GFP^+^ cells is shown. The results represent two independent experiments performed in duplicates. (D and E) Four days post chemical induction the virus supernatants of two K-DG75 wt and K-DG75 CBF1 ko cells (designated a and b) were harvested, concentrated and used to infect HEK293 cells. The HEK293 cells were cultivated in the absence or presence of puromycin for 48 h and analyzed for GFP expression by fluorescence microscopy and flow cytometry. (D) Phase contrast and fluorescence microscopy images of untreated and viral supernatant treated HEK293 cells. Numbers in the lower panels indicate the percentage of GFP positive HEK293 cells as determined by flow cytometry. (E) Viability of infected HEK293 cells was measured by flow cytometry using forward and sideward scatter. Numbers indicate the average percentage of living (black) and dead (red) cells as determined in 3 independent experiments. (F) Virion associated extracellular viral genome copy numbers of concentrated cell culture supernatants obtained from non-induced or induced BC-1, K-DG75 wt and K-DG75 CBF1 ko cells were determined by real-time PCR. Results are presented as mean values calculated from 2 independent experiments.

Next we examined if the virus can reactivate in K-DG75 cell lines. Upon treatment with sodium butyrate (NaB) the surrogate early lytic marker gene RFP was induced and expressed with similar frequencies in two independent K-DG75 wt and K-DG75 CBF1 ko cell lines (Fig. 2A). The combination of 12-O-Tetradecanoylphorbol-13-acetate (TPA) and NaB further enhanced RFP expression with a negligible difference between wt and CBF1 ko cells (Fig. 2B). Initiation of lytic reactivation thus does not appear to be a CBF1 dependent process in these B cells. We next analyzed if CBF1 proficient and deficient K-DG75 cell lines exhibit similar rates of viral replication upon lytic reactivation by TPA/NaB treatment. The rate of dead cells was determined for treated and untreated cell populations by trypan blue exclusion assays. The TPA/NaB treatment caused similar rates of dead cells in both populations (Fig. S1A). Viable subpopulations were defined by forward and sideward scatter signals (Fig. S1B) and RFP^+^/GFP^+^ cells were separated from RFP^−^/GFP^+^ cells by cell sorting. Equal numbers of sorted cells were compared for the number of intracellular viral genomes. Viral copy numbers increased in CBF1 proficient and deficient cells, but the increase in CBF1 proficient cells was two fold stronger (Fig. 2C).

Since lytic viral replication was impaired in CBF1 deficient B cells we investigated whether virus production was also attenuated. K-DG75 B cells were treated with TPA and NaB for 4 days, supernatants were harvested, concentrated 100-fold and used to infect HEK293 cells. These HEK293 cells were cultured either in the presence or absence of puromycin for 48 h and subsequently analyzed for GFP expression by fluorescence microscopy (Fig. 2D). In addition, cellular viability was monitored by flow cytometry using forward and sideward scatter to visualize viable and dead cell populations (Fig. 2E). GFP positive HEK293 cells were readily detected after infection with K-DG75 wt cell supernatants. K-DG75 CBF1 ko cells produced no or extremely low numbers of virions that conferred GFP expression. As a second marker for successful infection we tested whether the viral supernatants conferred puromycin resistance (Fig. 2D and E). Indeed, HEK293 cells infected with viral supernatants harvested from K-DG75 wt cells could be expanded in the presence of puromycin while HEK293 cells infected with supernatants from K-DG75 CBF1 ko cells lost their typical shape and their adherent phenotype and subsequently died (Fig. 2E). These results suggest that supernatants derived from CBF1 deficient K-DG75 cells contain no or only low amounts of infectious virus. To further confirm this assumption, extracellular viral genome copy numbers in K-DG75 wt and CBF1 ko derived supernatants were quantified and compared to virion DNA produced by BC-1. Virus production was induced ∼165-fold upon chemical reactivation of K-DG75 wt cells, approximately 3-fold higher than in BC-1 cells. In contrast, virion production by CBF1 deficient cells was barely detectable (Fig. 2F). In summary, our data show that K-DG75 cells can produce infectious virus. Genome replication was attenuated in CBF1 deficient K-DG75 cells and later processes required for morphogenesis of infectious virus were blocked.

### Lytic viral gene expression is severely attenuated in CBF1 deficient KSHV infected B cells

**Figure 3:**
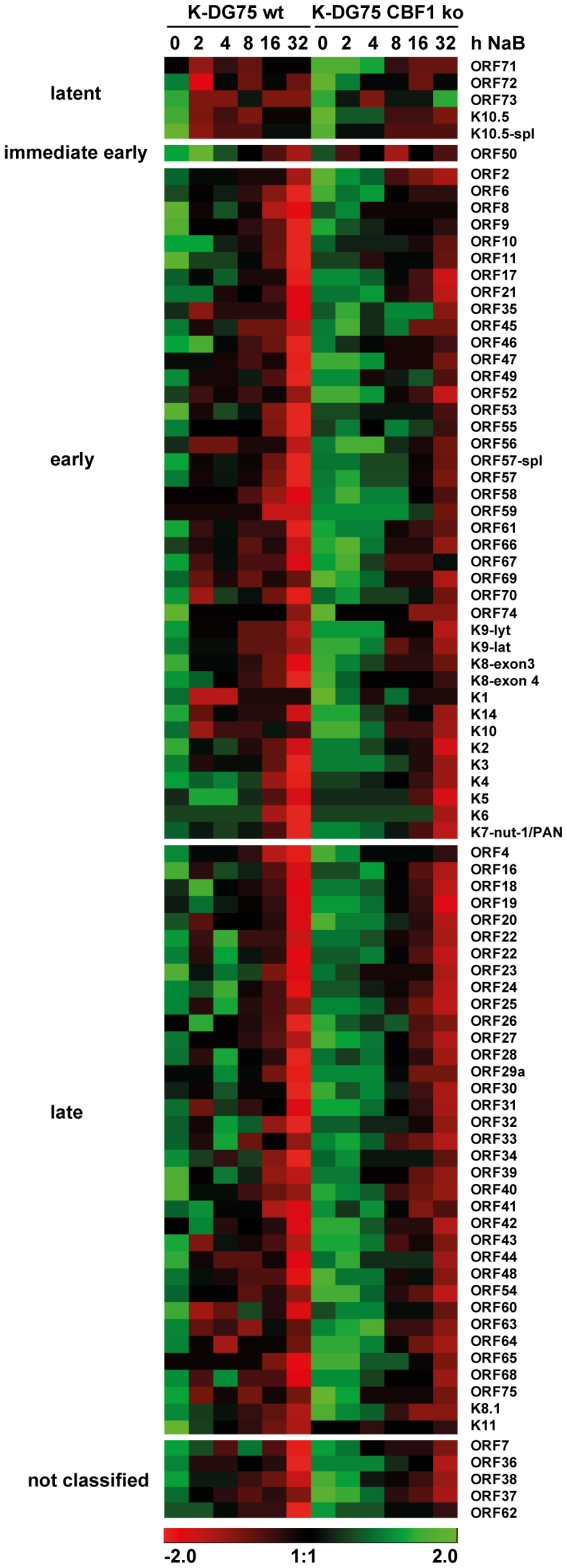
Genome-wide viral gene expression profiles of CBF1 proficient and deficient KSHV infected B cells. K-DG75 wt and K-DG75 CBF1 ko cells were treated with 3 mM NaB for 0, 2, 4, 8, 16, or 32 hours. Total RNA was harvested, enriched for the poly-adenylated fraction and transcribed into cDNA. Viral transcripts were quantified by real-time PCR and normalized to β-actin [51]. Viral gene expression patterns are shown as dCt values (Ct post lytic induction for individual time span - Ct prior lytic induction) in a heat map presentation after normalization to β-actin expression. Induction of viral genes results in negative dCt values. Negative dCt values represent high expression levels and are marked in red, intermediate expression levels are marked in black and positive values represent low expression levels and are marked in green. Vertical columns represent data obtained for serial time points post chemical induction for CBF1 proficient and deficient K-DG75 cells. Horizontal rows represent data for all tested KSHV genes. The heat map is split according to their classification into latent (n = 5), immediate early (n = 1), early (n = 40) and late (n = 35) genes and viral genes which have not yet been classified (n = 5) as reviewed [29].

In order to examine how the differences in viral reactivation were reflected by alterations in viral gene expression patterns we analyzed lytic viral gene expression in CBF1 proficient and deficient K-DG75 cells before and 2, 4, 8, 16 or 32 h post induction by NaB. The viral gene expression profile was analyzed using a previously developed real-time RT-PCR array for KSHV that represents 86 viral genes [27], [28]. The results of this genome wide analysis of viral reactivation in CBF1 proficient and deficient K-DG75 cells are shown as heat map (Fig. 3). The genes were arranged into groups of latent, early and late lytic genes as reviewed [29]. Induction of lytic viral genes could be observed in CBF1 proficient and deficient K-DG75 cells but was severely attenuated and delayed in CBF1 deficient cells. The difference between CBF1 proficient and deficient K-DG75 cells was most pronounced 32 h post induction.

**Figure 4:**
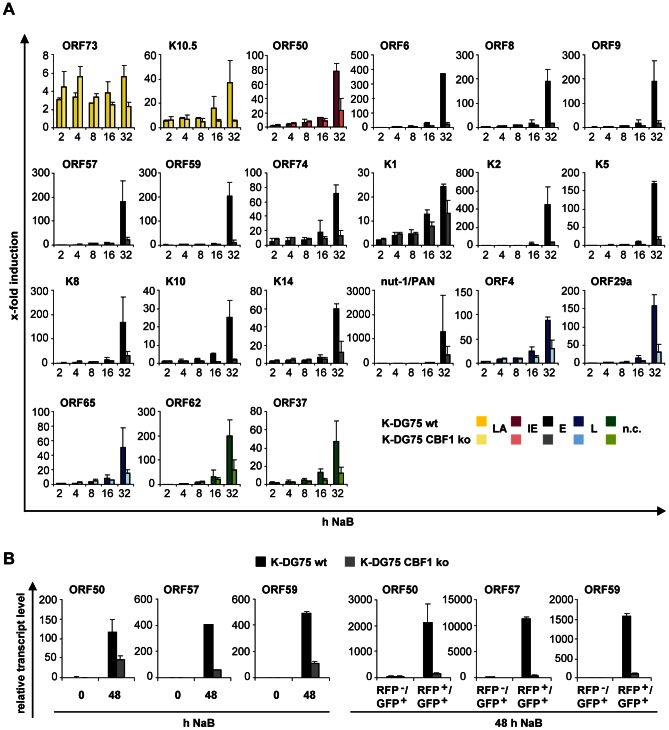
Confirmation of gene expression patterns for selected viral genes in CBF1 proficient and deficient K-DG75 cells. (A) The expression levels of 21 selected KSHV genes which are active in the latent state or different phases of the lytic cycle (ORF73/LANA, K10.5/vIRF3 (latent, LA), ORF50/RTA (immediate early, IE), ORF6, ORF8, ORF9, ORF57, ORF59, ORF74, K1, K2, K5, nut-1/PAN, K8, K10, K14 (early, E), ORF4, ORF29a, ORF65 (late, L), ORF37 and ORF62 (not classified, n.c.)) were determined by real-time RT-PCR analysis using primers distinct from the set of primers used for the genome-wide PCR array. Results were normalized to cellular β-actin expression and presented as x-fold increase post induction by 3 mM NaB for the indicated time periods. The results are shown as the mean values of 2 experiments performed with 2 independent cell lines. (B) K-DG75 wt and K-DG75 CBF1 ko cells were treated with 3 mM NaB for 48 h and relative transcript levels for ORF50/RTA, ORF57 and ORF59 were determined by real-time RT-PCR before and after treatment (left panels). A fraction of the chemically treated cells was separated into uninduced RFP^−^/GFP^+^ and induced RFP^+^/GFP^+^ populations and again transcript levels were determined (right panels). Two independent experiments were performed and mean values of duplicate PCR reactions of a representative experiment are shown.

A selection of viral genes was retested by real-time RT-PCR choosing primers distinct from those primers used for the array analysis (Fig. 4A). These results confirmed that all lytic genes were induced in CBF1 proficient and deficient K-DG75 cells but the degree of induction was different. Again the difference was best seen at late time points, when viral replication had already been initiated. The latent ORF73/LANA expression was not changed dramatically by chemical induction. Latent K10.5/vIRF3 was only induced in CBF1 proficient K-DG75 cells. As already seen by the array analysis, CBF1 dependent gene induction was not confined to a specific gene class. Thus, CBF1 appears to be required at multiple stages during the reactivation process.

Chemical induction of KSHV infected cells typically leads to reactivation of only a subset of the cellular population. We thus sorted RFP^−^/GFP^+^ latent and RFP^+^/GFP^+^ lytic populations and tested them for induction of the immediate early gene ORF50/RTA and the early lytic genes ORF57 and ORF59. As expected the difference in gene expression between CBF1 proficient and deficient K-DG75 cells was more pronounced if selected subsets were analyzed (Fig. 4B).

**Figure 5:**
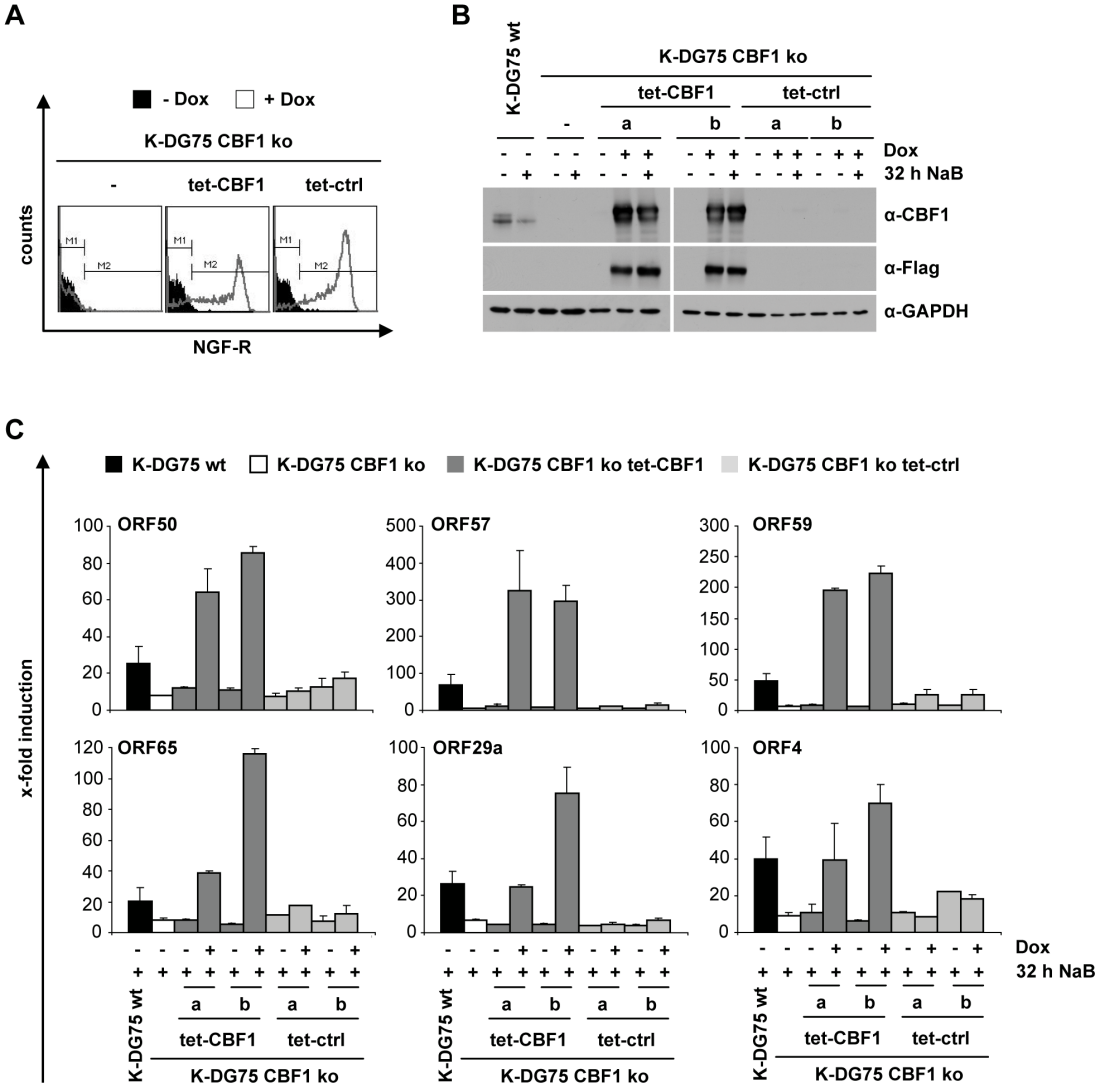
Ectopic expression of CBF1 rescues induction of lytic viral genes in K-DG75 CBF1 ko cells. K-DG75 CBF1 ko cells were stably transfected with an expression vector for Flag-tagged CBF1 (tet-CBF1) under the control of a bidirectional promoter, which allows the simultaneous expression of NGF-receptor (NGF-R) and Flag-CBF1, or transfected with a control vector (tet-ctrl). Stable cell lines were cultivated in the presence of doxycycline or left untreated. Untransfected (−) K-DG75 wt and CBF1 ko cells are shown for comparison. (A) The expression of NGF-R was monitored by flow cytometry. (B and C) The cells were treated with 3 mM NaB. (B) 30 µg of total cellular protein extract of K-DG75 wt or CBF1 ko cells or 10 µg of protein extract of doxycycline induced K-DG75 CBF1 ko tet-CBF1 and tet-ctrl cells were analyzed by immunoblotting using CBF1, Flag or GAPDH specific antibodies. (C) The transcript levels of ORF50/RTA, ORF57, ORF59, ORF29a, ORF65 and ORF4 were determined by real-time RT-PCR. Results are shown as x-fold induction compared to values obtained from cells not treated with NaB. Results are given as mean values for data obtained from 2 independent experiments.

In order to confirm our results we reintroduced a Flag-tagged version of the CBF1 protein into K-DG75 CBF1 ko cell lines. The expression construct, pRTS-2, we used carries a bidirectional doxycycline responsive promoter [30]. Induction of the gene of interest can be monitored by flow cytometry of a surrogate marker, a truncated version of the NGF-receptor, on the cell surface. Stable cell lines carrying the Flag-CBF1 expression construct showed doxycycline dependent NGF-receptor and Flag-CBF1 expression while cells carrying a control vector only expressed the NGF-receptor (Fig. 5A and B).

Next the induction of a series of lytic viral genes (ORF50/RTA, ORF57, ORF59, ORF65, ORF29a and ORF4) was tested in K-DG75 CBF1 ko cells in which Flag-CBF1 was induced by doxycycline. Two independent inducible cell lines were tested and compared to two control cell lines. Induction of lytic viral genes was restored by Flag-CBF1 and even exceeded the levels reached in K-DG75 wt (Fig. 5C). This observation indicates that the endogenous cellular CBF1 protein level is rate limiting for viral reactivation in DG75 cells.

**Figure 6:**
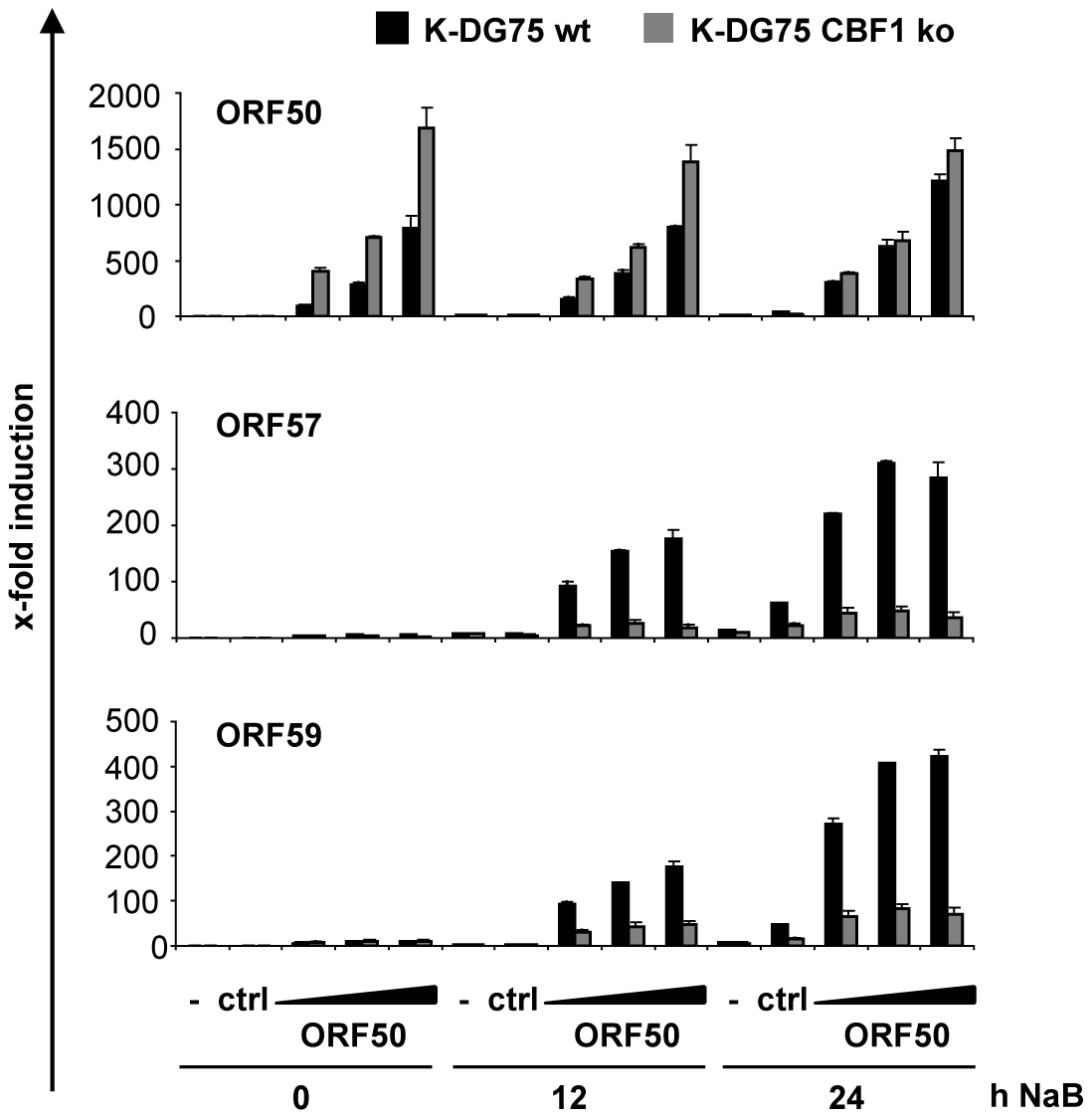
Ectopic expression of ORF50 does not rescue ORF57 and ORF59 expression in CBF1 deficient K-DG75 B cells. 1×10^7^ K-DG75 wt or K-DG75 CBF1 ko cells were transiently transfected with increasing amounts of an ORF50/RTA expression construct (15, 30 and 60 µg), the corresponding control vector (ctrl) or left non-transfected (−). 24 h post transfection cells were cultured with 3 mM NaB for 12 or 24 h. The transcript levels for ORF50/RTA, ORF57 and ORF59 were determined by real-time RT-PCR. Results are shown as x-fold induction compared to values obtained from non-transfected and uninduced cells. Three independent experiments were performed and mean values of duplicates of a representative experiment are shown.

ORF50/RTA is the major transactivator of ORF57 and ORF59. In addition, both, ORF57 and ORF59, are known to enhance RTA functions. ORF57 is a multifunctional protein which enhances splicing and translation efficiencies and supports the export of intronless viral mRNA and stabilizes transcripts [31]. ORF59 is one of the DNA polymerase processivity factors which are necessary for origin dependent viral replication [32]. Since ORF50/RTA induction was diminished in CBF1 deficient cells this defect in RTA expression could already cause a severe phenotype in CBF1 deficient DG75 cells. We hence wanted to ask if ORF50/RTA expression might rescue the deficiency of the CBF1 deficient K-DG75 cells. We thus expressed ORF50/RTA in K-DG75 cells and induced the lytic cycle by NaB. ORF50/RTA could strongly enhance ORF57 and ORF59 expression in NaB treated CBF1 proficient cells but induction in the absence of CBF1 was weak (Fig. 6). These results suggest that the lack of induction of ORF50 is not the only rate limiting factor for viral reactivation in CBF1 deficient B cells.

### ORF29a and ORF65 are novel CBF1 dependent target genes of RTA

**Figure 7:**
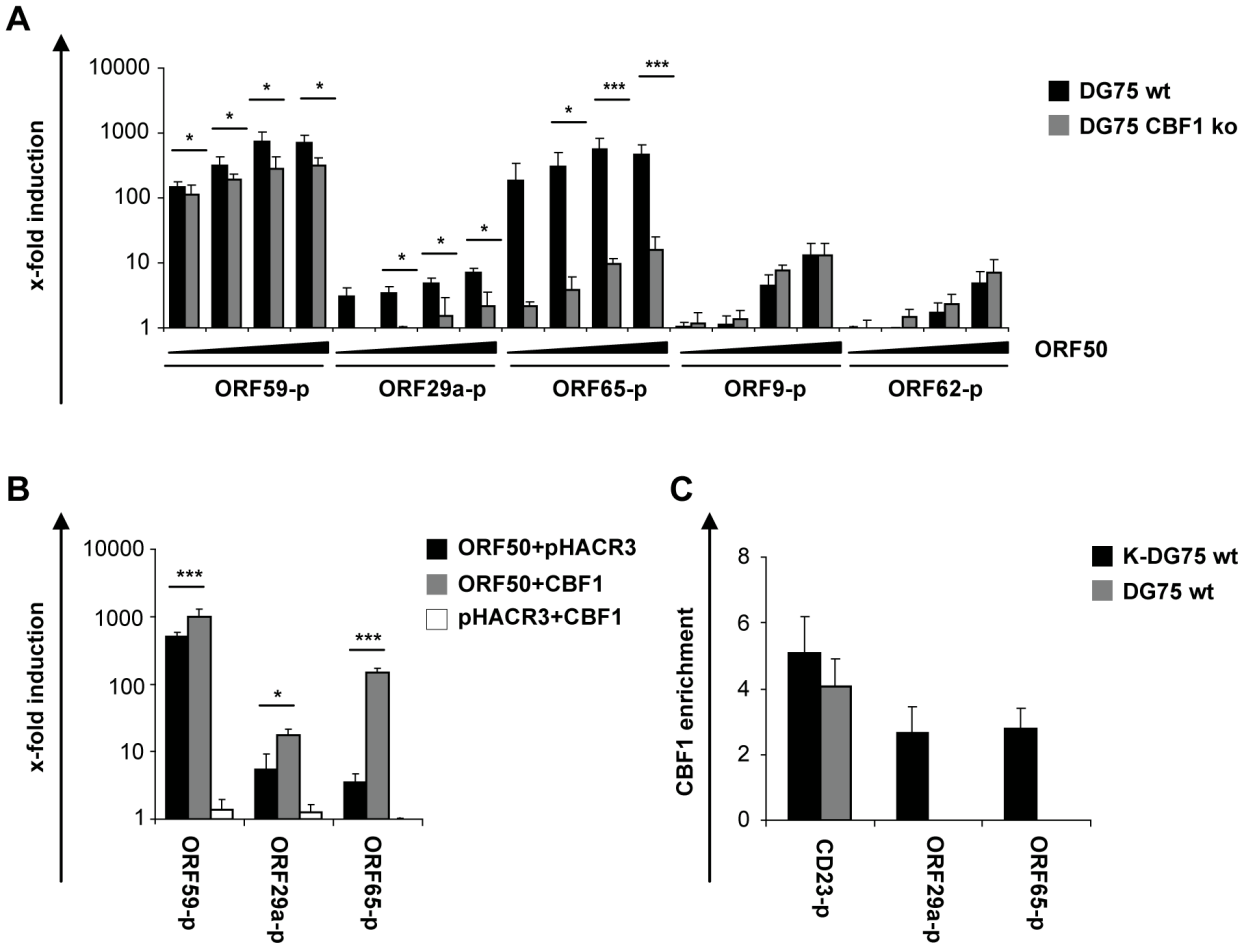
The late viral genes ORF29a and ORF65 are CBF1 dependent ORF50/RTA target genes. (A) 5×10^6^ KSHV negative DG75 wt or CBF1 ko cells were cotransfected with increasing amounts (0.05, 0.1, 0.5 and 1 µg) of ORF50/RTA expression or control vectors (pHACR3) and 3 µg of a luciferase promoter reporter construct containing a fragment of 1000 bp upstream of the translational initiation codon of the KSHV genes ORF59-p, ORF9-p, ORF29a-p, ORF62-p and ORF65-p. (B) KSHV negative DG75 CBF1 ko cells were cotransfected with 0.1 µg of a ORF50/RTA expression vector, 5 µg of a CBF1 expression vector or a control vector (pHACR3) and 3 µg of the ORF59, ORF29a-p and ORF65-p luciferase promoter reporter constructs. The ORF29a-p promoter constructs were cotransfected with 0.5 µg of the ORF50/RTA. The results in (A) and (B) represent means ± standard deviations derived from two independent experiments performed in triplicates. They are shown as x-fold induction compared to promoter activation by control vector and normalized to ß-galactosidase activity. The significance of changes of the promoter activities in the absence of CBF1 was calculated by student's t test (*p = 0.05–0.01, **p<0.01 or ***p<0.005). (C) Chromatin immunoprecipitation with CBF1 specific antibodies was performed to analyze CBF1 binding to promoters of the cellular CD23 gene, a well characterized CBF1 target, and the viral ORF29a and ORF65 promoters. As control, chromatin of KSHV negative DG75 cells was included. Co-immunopreciptiated DNA fragments were subsequently quantified by real-time PCR. Enrichment of CBF1 on specific genomic regions was calculated as percentage of the immunoprecipitated DNA compared to input DNA after subtraction of the isotype control signal and normalization to actin. The data represent the mean value of 3 independent experiments.

In summary, the analysis of viral gene expression profiles had shown that the induction of lytic genes of all classes was not blocked but attenuated in CBF1 deficient B cells. Viral genes that had been previously defined as genes which need CBF1 to recruit either RTA or Notch are listed in Table 1. In order to identify additional CBF1 dependent viral target genes the promoters of 4 selected genes which carried at least one CBF1 binding site predicted by using the MatInspector software of Genomatix were analyzed. Since the transcriptional start site of these genes had not been defined experimentally in the past a fragment of 1000 bp upstream of the translational start site was tested as a putative promoter. Transactivation of these putative promoter fragments by ORF50/RTA was tested in transient reporter gene assays using KSHV negative DG75 cells. For comparison the promoter of ORF59, a well known CBF1 dependent target gene, was included in the analysis. All promoters responded to ORF50/RTA even in the absence of CBF1 in a dose dependent manner indicating that there is no absolute requirement for CBF1 to recruit RTA to these target genes and activate transcription. Promoter activation of ORF59, ORF29a and ORF65 was significantly diminished in CBF1 deficient DG75 cells while transactivation of the ORF9 and ORF62 promoters was not impaired (Fig. 7A). By reintroducing CBF1, transactivation of the ORF59, ORF29a and ORF65 promoters was strongly enhanced confirming that CBF1 is a rate limiting factor for efficient activation of these promoters (Fig. 7B). Since we wanted to prove that CBF1 is recruited to the promoters of ORF29a and ORF65 we performed chromatin immunoprecipitations followed by real-time PCR for viral promoter fragments (Fig. 7C). While CBF1 could be readily detected on the promoter of its cellular target gene CD23 in KSHV positive and negative DG75 wt cells, as expected CBF1 was seen on viral promoters in KSHV infected DG75 wt cells only. In summary, these results suggest that the promoters of ORF29a and ORF65 are directly bound by CBF1.

## Discussion

The CBF1 protein is a DNA binding factor which is highly conserved in evolution. In mammals CBF1 is ubiquitously expressed in all tissues. Thus, CBF1 provides a central hub in every mammalian cell that is used by the cell to integrate information transmitted by external and internal stimuli. The two human γ-herpesviruses, EBV and KSHV, use CBF1 as a DNA adaptor for viral nuclear transcription factors to control the activity of viral and cellular target genes. EBV requires the CBF1 protein to enter and maintain the latent life cycle. In contrast, lytic infection and reactivation are CBF1 dependent processes in KSHV infected cells [8], [9]. RTA, the initiator of viral lytic replication, can bind to DNA directly but also uses CBF1 as an adaptor to regulatory elements of target genes. RTA expression is controlled by CBF1 binding sites within the RTA promoter. Thereby RTA initiates a positive feed back loop driving lytic reactivation [33]. On the other hand, LANA expression is also controlled by RTA via CBF1 dependent activation while LANA represses RTA expression via binding to CBF1 at the RTA promoter. However, subsequent studies using infective recombinant KSHV which lacked CBF1 binding sites in the RTA promoter demonstrated that these viruses had an enhanced capacity to infect and establish latency in primary human B cells in short term experiments [33]. In summary, CBF1 serves as the mediator of a negative and positive feed back loop that balances RTA and LANA expression and hence lytic and latent life cycle of the infected cell [34], [35]. Consequently, targeting CBF1 signaling by antiviral drugs could be highly attractive if critical stages of the virus life cycle relevant for pathogenesis could be efficiently blocked.

The functional analysis of CBF1 dependent processes has been hampered by the ubiquitous expression of CBF1 in human cells. Thus, the first report which described the interaction of RTA with CBF1 used CBF1 deficient fibroblasts derived from knock-out mice to study the contribution of CBF1 to establishment of latency, reactivation and lytic infection [9], [36]. While establishment of latency was not impaired in mouse fibroblasts reactivation was blocked at the stage of delayed early gene expression. In summary, our data confirm and extend the results of previous studies. Unlike murine fibroblasts, KSHV infected CBF1 deficient B cells can enter the lytic cycle with low efficiency but no specific block before the onset of viral replication or late gene expression is installed. Using the identical recombinant rKSHV.219 virus it has been described most recently that viral reactivation is blocked at multiple stages before viral DNA synthesis in CBF proficient murine fibroblasts [37]. These multiple blocks attenuated the induction of the lytic cascade in CBF1 deficient murine fibroblasts in the previous study and most likely explain why reactivation in human B cells can still be initiated.

The goal of our study was to identify CBF1 dependent processes that are rate limiting for viral reactivation and production of infectious viruses specifically in B cells. Both, CBF1 proficient and CBF1 deficient KSHV infected DG75 B cell lines could be established, carried similar numbers of intracellular viral genomes, and expressed similar amounts of the latent marker genes ORF73/LANA and K10.5/vIRF3.

During the first weeks post infection GFP expression was low but detected readily by flow cytometry and increased during the following weeks (Fig. 1). Since it was not relevant for the process of viral reactivation we have not analyzed the switch from GFP low to GFP high cultures in detail. At this point of our studies we do not want to exclude that GFP was transferred passively by viral particles and measured by flow cytometry early after infection. Future studies should reveal whether GFP expression levels changed during *in vitro* cell culture due to changes in viral genome copy numbers or were caused by epigenetic modifications of the viral chromatin. Since all KSHV infected DG75 cells were grown in selective media the infection process as well as establishment of the latent state in these cells may not reflect all features of the infection process under physiological conditions. Thus, at present the DG75 infection system cannot yet be used to study the potential role of CBF1 during early phases of the establishment of latency.

While CBF1 proficient and deficient B cell lines could induce the RFP reporter gene controlled by the early lytic PAN promoter of rKSHV.219, only CBF1 proficient DG75 B cells could produce infectious virus. Virus produced from K-DG75 wt cells induced a bright GFP signal but also conferred puromycin resistance to infected HEK293 cells (Fig. 2). Reactivation of the lytic cycle was initiated in both cell lines. However, activation of lytic genes was delayed in CBF1 deficient cells and did not reach the same expression levels as the CBF1 proficient cells 32 h post induction. Even 4 days after induction neither extracellular viral genomes nor release of infectious virus was detectable. Transcription of CBF1 dependent genes (ORF50/RTA, ORF57, ORF59, ORF65, ORF29a or ORF4) could be rescued or even enhanced by conditional expression of CBF1.

Thus, we could formally prove that the phenotype of CBF1 deficient K-DG75 cells is truly caused by the lack of CBF1. Ectopic expression of ORF50/RTA in K-DG75 CBF1 ko cells induced ORF57 and ORF59 expression weakly but induction rates never reached the levels obtained in K-DG75 wt cells. Thus, ORF50/RTA induction in K-DG75 CBF1 ko cells is essential but not the single rate limiting factor for reactivation. Our results suggest that the attenuated lytic gene expression levels are caused by additive effects of genes that can only be weakly activated by RTA in the absence of CBF1. While virus replication is still detected in K-DG75 CBF1 ko cells at reduced levels, virion production is entirely abolished. Interestingly, we could not identify a single gene that is not activated in K-DG75 CBF1 ko cells during reactivation and thus completely dependent on CBF1 for activation (Fig. 3). We conclude that the lack of CBF1 has pleiotropic effects during the reactivation process caused by direct and indirect effects triggered by ORF50/RTA or perhaps other viral CBF1 binding proteins like ORF73/LANA or vIRF4 [38], [39]. While LANA is an antagonist of RTA function, vIRF4 cooperates and enhances RTA activities [40]. Whether this cooperation of vIRF4 and RTA is CBF1 dependent still needs to be clarified.

As summarized in Table 1 there is a growing list of lytic viral genes requiring CBF1 for activation. In search for additional viral ORF50/RTA responsive and CBF1 dependent promoters, the putative promoters of ORF29a, ORF65, ORF9 and ORF62 were analyzed in reporter gene assays. The promoter of ORF59 was studied in parallel since ORF59 is a well characterized target gene of RTA (Fig. 7A). While activation of the endogenous ORF59 gene requires CBF1, ORF59 promoter activation is attenuated if CBF1 binding sites are deleted but RTA DNA binding sites are retained [9], [41]. Our experiments confirm that the requirement for CBF1 is much more pronounced if endogenous ORF59 gene expression is studied. These results might suggest that the chromatin state of the ORF59 gene has an important impact on promoter responses to RTA. If so, this could be relevant for the biology of the virus since the viral DNA in the infectious virus is chromatin free and unmodified.

While activation of the endogenous transcripts of ORF9 and ORF62 was CBF1 dependent, activation of the promoter reporter constructs of these genes by RTA was CBF1 independent. For ORF9 and ORF62 we cannot exclude that we have used incomplete promoter fragments which recruit RTA directly but do not carry the relevant CBF1 responsive elements. Perhaps these CBF1 binding sites are located in remote enhancers as it has been demonstrated recently for the CBF1 interaction partner Epstein-Barr virus nuclear antigen 2 in the context of the cellular genome [42]. Alternatively, the CBF1 dependent effects measured on endogenous gene expression were chromatin dependent, post-transcriptional, and caused by the attenuated lytic cascade in CBF1 deficient cells.

In contrast, RTA activation of the promoters of ORF29a and ORF65 was strongly impaired in the absence of CBF1 but could be rescued by ectopic expression of CBF1. In addition, CBF1 binding to these promoters could be demonstrated. Thus, ORF29a and ORF65 are RTA target genes which require direct binding of CBF1 to promoter sites. Both are late viral genes which are critical for viral morphogenesis and virus production.

For this study, we have developed a new cell culture model to study KSHV reactivation in human B cells. The DG75 human B cell line will be a versatile tool to study KSHV mutants in a cellular background that permits to inactivate genes by gene targeting. In the future individual KSHV loss of function mutants can be tested and these experiments can be combined with specific DG75 variants deficient for selected cellular proteins.

In summary, the results obtained with this novel B cell system strongly suggest that CBF1 signaling in human B cells has pleiotropic effects that coordinate and enhance the course of KSHV lytic viral gene induction at multiple stages. As exemplified by the two novel CBF1 dependent late genes ORF29a or ORF65 it appears that CBF1 is a global player, required at multiple stages to coordinate the lytic cascade. If antivirals targeting CBF1 signaling could be established we would expect that KSHV reactivation is severely impaired.

## Materials and Methods

### Cell lines and culture conditions

The cell lines BC-1 [1], BCBL-1 [22], HEK293 [43], DG75 wt [44], and DG75 CBF1 ko (SM224.9) [19] have been described. Routinely all cell lines were grown in RPMI 1640 supplemented with 10% fetal calf serum (FCS), penicillin (100 U/ml), streptomycin (100 µg/ml), and glutamine (4 mM) and maintained at 37°C in a 5% CO_2_ atmosphere. BC-1 and BCBL-1 were cultured in media containing 20% FCS. K-DG75 wt a (BS532.2a) and b (BS854.2b) and K-DG75 CBF1 ko a (BS532.1a) and b (BS648.1e) were cultured in media containing 20% FCS and puromycin (4 µg/ml). K-DG75 CBF1 ko tet-CBF1 a (BS1177.3) and b (BS1177.7) or tet-ctrl a (BS1247.8) and b (BS1247.9) were cultured in media containing 20% FCS, puromycin (4 µg/ml) and hygromycin B (400 µg/ml). Vero cells containing rKSHV.219 were kindly provided by J. Vieira (University of Washington) [20] and grown in DMEM supplemented with 10% FCS and 5 µg/ml puromycin.

### Plasmids

The Gateway compatible destination vectors pHACR3 [45] as well as the CBF1 expression vector (AJ247) have been published previously [19]. The vector pcDNA3.1-lacZ (Invitrogen) is commercially available. The pENTRY construct encoding ORF50/RTA has been published [46]. For expression in mammalian cells ORF50/RTA was transferred into destination vector pHACR3 by LR reaction (Invitrogen). A Triple-Flag-CBF1 ORF was cloned into pRTS-2 containing a hygromycin B resistance cassette and a bidirectional doxycycline (Dox)-regulated promoter which drives the simultaneous expression of a truncated NGF-receptor and Flag-CBF1 after addition of Dox [30]. In order to generate KSHV promoter constructs (ORF59-p, ORF9-p, ORF29a-p, ORF62-p and ORF65-p) fragments of 1000 bp upstream of the translational initiation codon of the KSHV genes were amplified by PCR using genomic DNA isolated from BC-1 cells as template and specific primers containing restriction sites. The PCR products were ligated into a luciferase reporter plasmid carrying a minimal silent promoter (Ga50-7). Primers used for cloning are listed in supplementary Table S1. The prediction of potential CBF1 binding sites in these KSHV promoter fragments was done by using the MatInspector software provided by Genomatix (matrices V$RBPJK.01 (cgTGGGaa) and V$RBPJK.02 (gTGGGaaa), core similarity 1.0 and matrix similarity >0.8). The position of the promoter fragments and the potential CBF1 binding sites in the viral genome are listed in supplementary Table S2.

### Induction of the lytic cycle, preparation and quantification of rKSHV.219 supernatant

For lytic cycle reactivation cells were treated with sodium butyrate (NaB) and/or 12-O-Tetradecanoylphorbol-13-acetate (TPA) as indicated. 1.5×10^5^ cells per ml were induced. Virus was harvested as described before [47]. Briefly, 4 days post induction cells were pelleted and the supernatant was filtered through a 0.45 µm pore-size filter. The virus was concentrated and washed twice in RPMI 1640 media without supplements by ultracentrifugation at 25,000 rpm in a Beckman SW28 rotor for 3 hours. Finally, the virus pellet was resuspended in 1/100 volume of the initial volume in cell culture medium. To determine the number of infectious virus HEK293 cells were infected with serial dilutions of the viral supernatants and the number of GFP positive cells was counted [20].

### Infection of DG75 wt or CBF1 ko cells with rKSHV.219 and puromycin selection

2×10^5^ DG75 wt or CBF1 ko cells per ml were seeded in a volume of 100 µl in a 96-well plate. The following day cells were infected with virus supernatant of Vero-rKSHV.219 cells with a multiplicity of infection (MOI) of factor 5. The culture plate was centrifuged at 300 g for 40 min at 32°C. One day post infection medium was replaced. 7 days post infection selection for rKSHV.219 positive cells was started by adding puromycin (1 µg/ml). Every 7 days half of the culture medium was replaced with medium supplemented with an increasing concentration of puromycin up to 4 µg/ml. Stable GFP positive cell lines were designated K-DG75 wt a (BS532.2a) and b (BS854.2b) or K-DG75 CBF1 ko a (BS532.1a) and b (BS648.1e).

### Dox-inducible Flag-CBF1 expression in K-DG75 CBF1 ko cells

K-DG75 CBF1 ko cells were transfected with the Triple-Flag-CBF1 pRTS-2 vector or a Triple-Flag control pRTS-2 vector by electroporation. Cells were selected in the presence of hygromycin B (400 µg/ml) and puromycin (4 µg/ml). Stable cell lines were designated K-DG75 CBF1 ko tet-CBF1 a (BS1177.3) and b (BS1177.7) or tet-ctrl a (BS1247.8) and b (BS1247.9).

### Immunofluorescence microscopy

Digital images were acquired using the Openlab acquisition software (Improvision) and a microscope (Axiovert 200 m, Carl Zeiss MicroImaging, Inc.) connected to a 5 charge-coupled device camera (ORCA-479, Hamamatsu).

### Flow cytometry and cell sorting

Infection of DG75 wt or CBF1 ko cells by rKSHV.219 or of HEK293 cells was monitored by GFP expression. NGF-receptor expression of K-DG75 CBF1 ko tet-CBF1 or tet-ctrl cells after Dox treatment was analyzed using a primary α-NGF-receptor antibody (HB8737-1, ATCC) or an isotype control and a Cy5-coupled secondary antibody (Dianova). Fluorescence of cells was detected and analyzed using a FACSCalibur system and CellQuest Pro software (BD Bioscience). To determine the percentage of lytically induced RFP^+^/GFP^+^ K-DG75 cells or to separate RFP^−^/GFP^+^ and RFP^+^/GFP^+^ K-DG75 cells, cells were sorted and analyzed using FACSAria III cell sorter (BD Bioscience) and FlowJo software (version 7.6.4).

### Isolation and quantification of intracellular and extracellular KSHV genomes

For determination of intracellular KSHV DNA copy numbers 1×10^6^ cells were harvested, washed in PBS, resuspended in solution A (10 mM Tris-HCl, pH 8.3, 100 mM KCl, 2.5 mM MgCl_2_) and lyzed in solution B (10 mM Tris-HCl, pH 8.3, 2.5 mM MgCl_2_, 1% Tween 20, 1% NP-40) supplemented with RNase A (0.2 µg/µl) and Proteinase K (1.5 µg/µl) and incubated for 30 min at 37°C and subsequently for 60 min at 50°C. DNA was purified by phenol-chloroform extraction. Extracellular virion-associated KSHV genomes in culture supernatants of lytically induced cells were isolated as described [48]. Intracellular and extracellular viral DNA was analyzed by real-time PCR as described below. For quantification a standard curve with defined numbers of PCR fragments of the KSHV genome corresponding to the ORF50 promoter region and β-actin was generated and analyzed in parallel. The intracellular KSHV copy number per cell was determined after normalization to β-actin. Primers used for real-time PCR are listed in supplementary Table S3.

### Analysis of viral gene expression by a KSHV real-time PCR Array

K-DG75 wt or CBF1 ko cells were lytically induced with 3 mM NaB for 0, 2, 4, 8, 16 or 32 hours. RNA of was extracted, mRNA was enriched and cDNA was synthesized by reverse transcription as described [27]. The KSHV real-time PCR Array was performed in collaboration with D. Dittmer (Lineberger Comprehensive Cancer Center, Chapel Hill) as described previously [27]. dCt values of each primer pair of 86 KSHV genes were normalized to β-actin. Heat map representation of the viral gene expression profile was generated using the software Genesis [49].

### Relative quantification of viral transcripts by real-time RT-PCR

RNA of 5×10^6^ cells was extracted, treated with DNase and cDNA was synthesized using the High Capacity cDNA Reverse Transcription Kit (Applied Biosystems) according to the manufacturer's protocol. Relative quantification of the transcripts by real-time PCR was performed with the LightCycler 480 II system and the data were processed by LightCycler 480 software, version 1.5.0.39 (Roche). cDNA was amplified using the LightCycler 480 SYBR Green I Master mix according to the manufacturer's protocol (Roche). Cycling conditions were 1 cycle of 95°C for 10 min and 45 cycles of denaturation (95°C for 2 s), annealing (63°C for 10 s), and extension (72°C for 20 s). All PCR products were examined by melting curve analysis and the expected PCR fragment size was verified by agarose gel electrophoresis. To account for differences in reaction efficiencies, a standard curve was generated for each primer pair by using the serial dilutions of PCR products as templates for amplification and plotting the crossing points versus the known dilutions. All data were normalized to the relative abundance of the β-actin transcript. Primers used for real-time RT-PCR are listed in supplementary Table S3.

### Transfection of cells and reporter gene assays

1×10^7^ DG75 wt or CBF1 ko cells were transfected with the indicated plasmid DNA by electroporation (220 V, 950 µF) using a gene Pulser II (Bio-Rad). Luciferase reporter gene assays were performed as described previously [39]. Briefly, cells were transfected with 3 µg luciferase reporter construct, 1 µg pcDNA3.1-lacZ and the indicated expression plasmid. The DNA amounts were adjusted by adding the corresponding empty vector. Cells were harvested 48 h after transfection and luciferase and β-galactosidase activity was measured. Transfections were done in triplicates and results were normalized to ß-galactosidase activity derived from the reporter construct pcDNA3.1-LacZ included in each sample.

### Western blot analysis

Western blot analysis was performed as described [19]. The α-CBF1 rat monoclonal antibody RBP-7A11 (produced in collaboration with E. Kremmer, Helmholtz Center Munich) has been published [19]. The α-Flag (Sigma-Aldrich) and α-GAPDH (Millipore) antibodies were purchased. .

### Chromatin immunoprecipitation (ChIP) analysis

ChIP analysis was performed as described [50] with minor modifications using a mixture of hybridoma supernatant of the α-CBF1 rat monoclonal antibodies RBJ-1F1 and RBJ-6E7 (see supplementary text and Table S4 for details).

## Supporting Information

Figure S1 Cell death rates of NaB/TPA treated CBF1 proficient and deficient K-DG75 cells. K-DG75 wt and K-DG75 CBF1 ko cells were treated with increasing concentrations of NaB/TPA for 32 h. (A) Dead cells were identified by trypan blue staining and counted. The results are given as the mean percentage of dead cells from two independent experiments analyzed in triplicates. (B) Forward/sideward scattering of treated and untreated cells was monitored by FACS analysis. The gates indicate the homogenous and viable cell populations that were used for isolating RFP^+^/GFP^+^ cells for the experiments described in Figure 2.
(TIF)

Text S1 Chromatin immunoprecipitation (ChIP) analysis.
(DOC)

Table S1 Primers used for generation of luciferase reporter gene constructs.
(DOC)

Table S2 Localization of the KSHV promoter fragments and the predicted CBF1 binding sites corresponding to the BC-1 genome (PEL, NCBI accession no. NC_U75698).
(DOC)

Table S3 Primers used for real-time RT-PCR and quantification of KSHV copy numbers.
(DOC)

Table S4 Primers used for real-time PCR of ChIP DNA.
(DOC)
