# Supplementary figures and images for: Abortive Lytic Reactivation of KSHV in CBF1/CSL Deficient Human B Cell Lines
Source: PLoS Pathog. 2013 May 16;9(5):e1003336. doi: 10.1371/journal.ppat.1003336 (PMC3656114; doi:10.1371/journal.ppat.1003336)

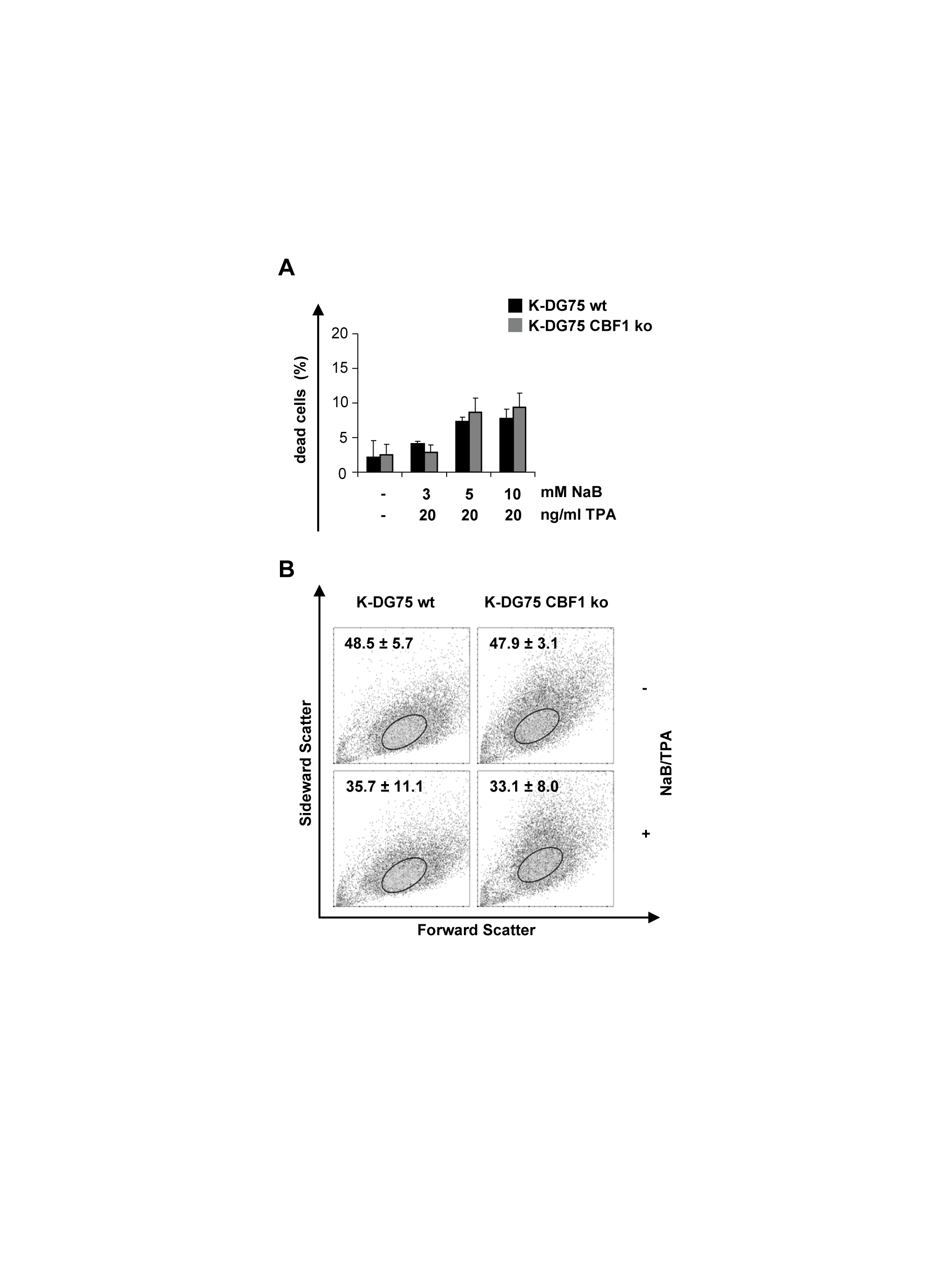

Supplement: Figure S1 — Cell death rates of NaB/TPA treated CBF1 proficient and deficient K-DG75 cells. K-DG75 wt and K-DG75 CBF1 ko cells were treated with increasing concentrations of NaB/TPA for 32 h. (A) Dead cells were identified by trypan blue staining and counted. The results are given as the mean percentage of dead cells from two independent experiments analyzed in triplicates. (B) Forward/sideward scattering of treated and untreated cells was monitored by FACS analysis. The gates indicate the homogenous and viable cell populations that were used for isolating RFP+/GFP+ cells for the experiments described in Figure 2. (TIF) [file ppat.1003336.s001.tif]
