# Supplementary material for: Abortive Lytic Reactivation of KSHV in CBF1/CSL Deficient Human B Cell Lines
Source: PLoS Pathog. 2013 May 16;9(5):e1003336. doi: 10.1371/journal.ppat.1003336 (PMC3656114; doi:10.1371/journal.ppat.1003336)
Supplement: Text S1 — Chromatin immunoprecipitation (ChIP) analysis. (DOC) [file ppat.1003336.s002.doc]

**Supplementary text S1**

**Chromatin immunoprecipitation (ChIP) Analysis**

For ChIP analysis 4 x 10^7^ K-DG75 wt cells were harvested, washed twice in ice cold PBS and resuspended in 20 ml RPMI1640 without supplements. After treating cells with 1% formaldehyde for 4 min at room temperature (RT), glycine was added to a final concentration of 125 mM for 5 min at RT. Cells were pelleted (500 g, 5 min, 4°C) and washed twice in PBS. To isolate the nuclei cells were resuspended 3 x in 10 ml ice cold lysis buffer (10 mM Tris-HCl, pH 7.5, 10 mM NaCl, 3 mM MgCl_2_, 0.5% NP-40) and centrifuged (300 g, 10 min, 4°C). The pellet was resuspended in 2 ml ice cold SDS lysis buffer (50 mM Tris-HCI, pH 8.1, 10 mM EDTA, pH 8.0, 1% SDS, 1 × protease inhibitor cocktail (PIC, Roche)), chromatin was fragmented by sonication using a Bioruptor (Diagenode). After centrifugation at 25.000 g for 10 min at 4°C aliquots of the supernatant were stored at -80°C. For immunoprecipitation 120 µl of the chromatin solution (2.4 x 10^6^ cell equivalents) were adjusted to a volume of 1.2 ml with dilution buffer (20 mM Tri-HCI, pH 8.0, 2 mM EDTA, pH 8.0, 1% Triton X-100, 150 mM NaCl, 1 x PIC) and incubated with 100 µl of a mixture of hybridoma supernatant of the α-CBF1 rat monoclonal antibodies RBPJ-1F1 and RBJ-6E7 (produced in collaboration with E. Kremmer, Helmholtz Center Munich) or the corresponding isotype control antibody overnight at 4°C by rotating. Protein G coupled sepharose beads were added for 3 hours at 4°. Immunoprecipitates were washed twice with ice cold wash buffer I (20 mM Tris-HCI, pH 8.0, 2 mM EDTA, pH 8.0, 1% Triton X-100, 150 mM NaCl, 0.1% SDS), once with wash buffer II (20 mM Tris-HCI, pH 8.0, 2 mM EDTA, pH 8.0, 1% Triton X-100, 500 mM NaCl, 0.1% SDS), once with wash buffer III (10 mM Tris-HCI, pH 8.0, 2 mM EDTA, pH 8.0, 250 mM LiCl, 1% NP-40, 1% sodium deoxycholate) for 5 min and twice with TE buffer. After every step beads were centrifuged at 2000 g for 2 min and in all buffers 1 x PIC was included. To elute DNA beads were resuspended in elution buffer (25 mM Tris-HCI, pH 7.5, 10 mM EDTA, pH 8.0, 0.5% SDS) and incubated at 65°C for 15 min. DNA supernatant was treated with RNase A (0.2 µg/µl) for 30 min at 37°C. The crosslink was reversed by Proteinase K (1.5 µg/µl) treatment for 1 hour at 42°C and further incubation over night at 65°C. The DNA was purified by phenol-chloroform extraction. For input DNA 1/10 of the chromatin solution used for immunoprecipitation was treated in parallel as described above. Input and ChIP DNA was analyzed by real-time PCR using standard curves for each primer pair. The enrichment of CBF1 on specific genomic regions was calculated as the percentage of immunoprecipitated DNA compared to input DNA after subtraction of the isotype control signal and after normalization to actin. The primers used for real-time PCR are listed in supplementary Table S4.
