# Supplementary material for: Abortive Lytic Reactivation of KSHV in CBF1/CSL Deficient Human B Cell Lines
Source: PLoS Pathog. 2013 May 16;9(5):e1003336. doi: 10.1371/journal.ppat.1003336 (PMC3656114; doi:10.1371/journal.ppat.1003336)
Supplement: Table S1 — Primers used for generation of luciferase reporter gene constructs. (DOC) [file ppat.1003336.s003.doc]

**Table S1: Primers used for generation of luciferase reporter gene constructs**

| **Construct** | **Primer** | **Sequence (5´-3´)** |
| --- | --- | --- |
| ORF59-p (CK6) | BS947fw-XhoI | GATCTCGAGGATTGCGGCCGTAGACGC |
|  | BS947rev-HindIII | ATCAAGCTTTTGGCGCTAACGCGCGAG |
| ORF9-p (ME2.4) | BS998fw-HindIII | CGTAAGCTTGGTGACGTTTAAGTTTTTGAAC |
|  | BS998rev-XhoI | CAGACTCGAGGATCTGCGGACGGTAATTTG |
| ORF29a-p (CK7) | BS948fw-XhoI | GATCTCGAGGTTTGCTTTGAGCTCGCTC |
|  | BS948rev-HindIII | ATCAAGCTTGACTTAATAAACTCTCTTTTAGA |
| ORF62-p (ME2.8) | BS1002fw-XhoI | GATCTCGAGATCCACGCCGGCAATGGA |
|  | BS1002rev-BamHI | CAGGGATCCGCTAGTAGCGGTATCTAGGT |
| ORF65-p (ME2.7) | BS1001fw-XhoI | GATCTCGAGCTTCCACACAGGCGGGC |
|  | BS1001rev-HindIII | ATCAAGCTTGTGCCCAGGCCGCCGA |
