# Supplementary material for: Abortive Lytic Reactivation of KSHV in CBF1/CSL Deficient Human B Cell Lines
Source: PLoS Pathog. 2013 May 16;9(5):e1003336. doi: 10.1371/journal.ppat.1003336 (PMC3656114; doi:10.1371/journal.ppat.1003336)
Supplement: Table S2 — Localization of the KSHV promoter fragments and the predicted CBF1 binding sites corresponding to the BC-1 genome (PEL, NCBI accession no. NC_U75698). (DOC) [file ppat.1003336.s004.doc]

**Table S2: Localization of the KSHV promoter fragments and the predicted CBF1 binding sites corresponding to the BC-1 genome (PEL, NCBI accession no. NC_U75698)**

| **Gene** | **Localization** | **Potential CBF1 binding site** | **Sequence** |
| --- | --- | --- | --- |
| ORF59 | 96740-97739 | 97662-97674 | cacgTGGGaaaca |
|  |  | 97601-97613 | ttttTGGGaatgg |
|  |  | 96985-96997 | aataTGGGaggaa |
| ORF9 | 10363-11362 | 11069-11081 | ctgcTGGGaatgc |
| ORF29a | 54677-55676 | 55255-55267 | cgttTGGGagcgt |
|  |  | 55427-55439 | ggtgTGGGaaatg |
|  |  | 55590-55602 | acgaTGGGatgat |
| ORF62 | 101195-10219 | 101363-101375 | cagtTGGGcaaca |
|  |  | 101714-101726 | atacTGGGcagct |
|  |  | 101758-101770 | gaagTGGGagggt |
| ORF65 | 112444-113443 | 112570-112582 | gcctTGGGacgcc |
|  |  | 112807-112819 | ttggTGGGaggaa |
|  |  | 113352-113364 | gagtTGGGcacag |
|  |  | 113391-113403 | cgtaTGGGcaaaa |
|  |  | 113433-113445 | ggccTGGGcacgg |
