# Supplementary material for: Abortive Lytic Reactivation of KSHV in CBF1/CSL Deficient Human B Cell Lines
Source: PLoS Pathog. 2013 May 16;9(5):e1003336. doi: 10.1371/journal.ppat.1003336 (PMC3656114; doi:10.1371/journal.ppat.1003336)
Supplement: Table S3 — Primers used for real-time RT-PCR and quantification of KSHV copy numbers. (DOC) [file ppat.1003336.s005.doc]

**Table S3: Primers used for real-time PCR and RT-PCR**

| **Gene** | **Primer** | **Sequence (5´-3´)** |
| --- | --- | --- |
| beta-actin | BS1269fw | TCACCCACACTGTGCCCATCTACGA |
|  | BS1269rev | CAGCGGAACCGCTCATTGCCAATGG |
| ORF50/RTA | BS554fw | CTGACGTCATGTCACCCTTG |
|  | BS554rev | TCTCTACACGGCACACCTTG |
| ORF50-promoter | BS729fw | GAATGCCACAATCTGTGCCCTCCAGCTCTCAC |
|  | BS729rev | GTGGCTGCCTGGACAGTATTCTCACAACAGAC |
| ORF57/Mta | BS551fw | AAGCGTACTGGGTGAGTTGC |
|  | BS551rev | TACTAAGCGGTTTCCCATCG |
| K10/vIRF4 | BS556fw | TGTGTATGGGAACCTTCGTG |
|  | BS556rev | TTACCAAACCACCAGCCTTC |
| K14 | BS613fw | ACGCCTCTTCTACCCAGGAG |
|  | BS613rev | TAGGCCCACCAGAGTAATGG |
| ORF74 | BS617fw | GCATGTCAGAACCGTGTCAG |
|  | BS617rev | GTCGCCTTAGCAGAGTGTCC |
| ORF59 | BS623fw | ACAGTCACCGTTTGGTCCTC |
|  | BS623rev | TGTACTCGACGCTGGCATAG |
| K1 | BS1002fw | TGATTTCAACGCCTTACACG |
|  | BS1002rev | GTTTCATTTCGTCCGTTTGG |
| ORF4 | BS1003fw | AAATCCTGGACGTTTGCATC |
|  | BS1003rev | AAGGCGTAGCATCATCTTGG |
| K10.5/vIRF3 | BS687fw | TCCTCAGATTCCGCGCC |
|  | BS687rev | TCACCTACACAGTGGGTCATCAC |
| ORF6 | BS1004fw | AGGACGTTAGCACGGTCATC |
|  | BS1004rev | TGCGTGCCAAACTTCTAGTG |
| ORF8 | BS1005fw | CGCCATCACCAACAAGTATG |
|  | BS1005rev | CACCCAGTGACGTGACAAAG |
| ORF9 | BS1006fw | TTCCACCCACCTACTTCCAG |
|  | BS1006rev | AAACGTTCACGCAGACACTG |
| ORF62 | BS1007fw | CCTTTATCATGGCCACAACC |
|  | BS1007rev | AGGTCCAATGGTCCAGAGTG |
| ORF65 | BS1008fw | CGTGGATGAGAGGGTTGTG |
|  | BS1008rev | ATATGTCGCAGGCCGAATAC |
| ORF29a | BS1009fw | GGGTCGCGTGTAGATTATGG |
|  | BS1009rev | CCAAGGAGACACGGAAAGAC |
| K5 | BS1011fw | GTTATCTTCGTTCGCGGTTG |
|  | BS1011rev | AGTGGCGTAGTCGCCTTAAC |
| nut-1/PAN | BS1012fw | CGGGTTATTGCATTGGATTC |
|  | BS1012rev | CAAAGTGGCCCGATTTACAC |
| K8 | BS1013fw | ATTTCGCAACAGCTTCCAAC |
|  | BS1013rev | TACCTGCTGCAGCTGTCTTG |
| ORF73/LANA | BS1035fw | TTGGATCTCGTCTTCCATCC |
|  | BS1035.1rev | ACCAGACGATGACCCACAAC |
| K2/vIL6 | BS1034fw | TCGTTGATGGCTGGTAGTTTC |
|  | BS1034rev | CGGTTCACTGCTGGTATCTG |
| ORF37/SOX | BS1033fw | CTTCAAAGCCAACCTCTTCG |
|  | BS1033rev | GTCCTTCGCACTTCTTGACC |
