# Supplementary material for: Abortive Lytic Reactivation of KSHV in CBF1/CSL Deficient Human B Cell Lines
Source: PLoS Pathog. 2013 May 16;9(5):e1003336. doi: 10.1371/journal.ppat.1003336 (PMC3656114; doi:10.1371/journal.ppat.1003336)
Supplement: Table S4 — Primers used for real-time PCR of ChIP DNA. (DOC) [file ppat.1003336.s006.doc]

**Table S4: Primers used for real-time PCR of ChIP DNA**

| **Gene** | **Primer** | **Sequence (5´-3´)** |
| --- | --- | --- |
| beta-actin | BS1380fw | CCACAGCCAGAGGTCCTCAG |
|  | BS1380rev | AGGAGCTCTTGGAGGGCATG |
| ORF29-promoter | BS1386fw | AGCACTGCGGTTACCCGG |
|  | BS1201rev | TGTCCCGGAAAAATCTCCGT |
| ORF65-promoter | BS1389fw | AGCAGTCCGCGCACGTCA |
|  | BS1389rev | CCCTTGCCATTCGAGACCT |
| CD23-promoter | KG572fw | GCCGTCCTTCTAACCCAAGAG |
|  | KG572rev | CCTGTGGGAACTTGCTGCTT |
